# Clear Cell Adenocarcinoma of the Urinary Bladder Is a Glycogen-Rich Tumor with Poorer Prognosis

**DOI:** 10.3390/jcm9010138

**Published:** 2020-01-03

**Authors:** Zhengqiu Zhou, Connor J. Kinslow, Peng Wang, Bin Huang, Simon K. Cheng, Israel Deutsch, Matthew S. Gentry, Ramon C. Sun

**Affiliations:** 1 Department of Molecular and Cellular Biochemistry, University of Kentucky College of Medicine, Lexington, KY 40536, USA; zhengqiu.zhou@uky.edu (Z.Z.); matthew.gentry@uky.edu (M.S.G.); 2 Department of Radiation Oncology, Vagelos College of Physicians and Surgeons, Columbia University Irving Medical Center, New York, NY 10032, USA; cjk2151@cumc.columbia.edu (C.J.K.); sc3225@cumc.columbia.edu (S.K.C.); id2182@cumc.columbia.edu (I.D.); 3 Division of Medical Oncology, Department of Internal Medicine, College of Medicine, University of Kentucky, Lexington, KY 40536, USA; p.wang@uky.edu; 4 Department of Biostatistics, College of Public Health, University of Kentucky, Lexington, KY 40536, USA; bhuang@kcr.uky.edu; 5 Herbert Irving Comprehensive Cancer Center, Vagelos College of Physicians and Surgeons, Columbia University Irving Medical Center, New York, NY 10032, USA; 6 Markey Cancer Center, University of Kentucky, Lexington, KY 40536, USA; 7 Department of Neuroscience, University of Kentucky College of Medicine, Lexington, KY 40536, USA

**Keywords:** glycogen, clear-cell adenocarcinoma, urinary bladder, SEER program database

## Abstract

Clear cell adenocarcinoma (CCA) is a rare variant of urinary bladder carcinoma with a glycogen-rich phenotype and unknown prognosis. Using the National Cancer Institute’s surveillance, epidemiology, and end results (SEER) program database, we documented recent trends in incidence, mortality, demographical characteristics, and survival on this rare subtype of urinary bladder cancer. The overall age-adjusted incidence and mortality of CCA was 0.087 (95% confidence interval (CI): 0.069–0.107) and 0.064 (95% CI: 0.049–0.081) respectively per million population. In comparison to non-CCAs, CCAs were more commonly associated with younger age (<60 years old, *p* = 0.005), female (*p* < 0.001), black ethnicity (*p* = 0.001), grade III (*p* < 0.001), and higher AJCC 6th staging (*p* < 0.001). In addition, CCA patients more frequently received complete cystectomy (*p* < 0.001) and beam radiation (*p* < 0.001) than non-CCA patients. Our study showed a poorer prognosis of CCAs compared to all other carcinomas of the urinary bladder (*p* < 0.001), accounted for by higher tumor staging of CCA cases. This study adds to the growing evidence that glycogen-rich cancers may have unique characteristics affecting tumor aggressiveness and patient prognosis. Additional mechanistic studies are needed to assess whether it’s the excess glycogen that contributes to the higher stage at diagnosis.

## 1. Introduction

Glycogen, a multibranched polymer of glucose, serves as our body’s main form of carbohydrate storage [1]. In the past decade, glycogen has become well-established that, in addition to its role in maintaining metabolic homeostasis in normal cells, it also has a crucial role in promoting tumor growth, especially under adverse conditions [2]. Under hypoxic conditions, which are commonly encountered by tumors cells, expression of transcription factor HIF1α increases glycogen accumulation [3]. Cancer cells have been shown to mobilize this excess glycogen via a p38α mitogen-activated protein kinase pathway to fuel cellular proliferation and metastasis [4]. Glycogen has also been proposed to maintain the Warburg effect in tumor cells, providing a mechanism for survival during nutrient deprivation [5]. Furthermore, glycogen’s inability to metabolize glycogen through small molecule inhibitors was able to induce apoptosis or senescence in tumor cells [6,7]. Altogether, cancer cells utilize glycogen as a way to alter its metabolic programing in order to adapt to the adverse tumor microenvironment and maintain tumor growth. 

Aberrant glycogen deposits have been identified in tumors from multiple origins, including cancers of the breast, kidney, uterus, lung, head and neck, bladder, ovary, skin, brain and colorectal tumors [8,9,10,11,12]. They are often identified as “clear cell” due to the transparent and ovoid appearance seen on histological staining. A poorer prognosis has been documented in clear cell carcinomas of the kidney [13], uterus [14], ovaries [15] and breast [16]. However, due to the rarity of some these tumors, the prognostic implications in other types of “clear cell” cancers remain unclear.

Clear cell adenocarcinoma of the urinary bladder (CCA) is a rare histological growth pattern first reported by Dow and Young in 1968 [17]. These tumors contain sheets of uniform ovoid cells with clear cytoplasm containing abundant glycogen [18,19]. Since there are no distinguishing symptoms of CCA, diagnosis is based on histopathological identification of these characteristics. Due to its rarity, information on the characteristics and prognosis of CCA have been limited to case reports, with less than 50 cases reported to date [19,20,21]. The largest existing literature review was performed by Lu et al., consisting of 38 case reports [21]. The review supported surgical resection as initial treatment for CCA and noted a possible increase in metastasis risk compared to urothelial carcinomas. However, the study determined that the prognosis of CCA was unclear as longer follow up periods were needed to more accurately assess survival characteristics [21]. No incidence and mortality data have been reported yet. 

As the first large-scale study to date, we utilized the National Cancer Institute’s surveillance, epidemiology, and end results (SEER) program database to conduct a retrospective assessment of incidence, mortality, demographics, and survival for CCA. Based on the previous literature that has shown a link between glycogen rich tumors and tumor aggressiveness [13,14,15,16], our study aimed to assess whether similar prognostic outcomes exist for CCAs. Using 91 cases of CCA and 205,106 cases of other urinary bladder cancers (non-CCA) obtained from the SEER Program database, we identified a poorer prognosis attributed to higher staging at time for diagnosis for CCAs. Our study contributes to the growing body of evidence revealing a possible link between glycogen and tumor aggressiveness. 

## 2. Experimental Section

### 2.1. Data Source

The SEER Program is the National Cancer Institute’s authoritative source of information on cancer incidence and survival capturing approximately 34.6% of the US population [22]. It is populated with high quality population-based data from national cancer registries. Vital status is updated annually and routinely undergoes quality-control checks.

### 2.2. Sample Selection and Coding

Age-adjusted incidence and mortality rates were calculated using the SEER*Stat Software (Version 8.3.6, National Cancer Institute, Bethesda, MD, USA) using all 91 cases of malignant cases of CCA of the urinary bladder and 205, 106 cases of non-CCA from 2004 to 2015 from the SEER Program database [23,24]. Incidence and mortality were age-adjusted by standardizing to the 2000 United States Census population. All other data collection and analysis were conducted as described previously [16,25]. We obtained the November, 2015 submission [26] and November, 2017 submission [27] from the SEER Program database and merged all identified cases of malignant cancers of the bladder identified by International Classification of Diseases-O-3 (ICD-O-3) codes C67.0–C67.9 from January 2004 to December 2015. Carcinomas of the bladder were determined based on the adapted classification scheme for adolescents and young adults. Cases of clear cell adenocarcinoma were identified by ICD-O-3 code 8310.

The following variables were collected and coded: AYA site recode, primary site, ICD-O-3 histology, age at diagnosis, sex, race, grade, American Joint Commission on Cancer (AJCC) 6th Edition Staging, AJCC 6th Edition TNM system, survival months, vital status, bone metastasis at diagnosis, brain metastasis at diagnosis, liver metastasis at diagnosis, lung metastasis at diagnosis, surgery, and radiation. Cases of AJCC 6th stage 0a and 0is were merged and referred to as “stage 0”. Ta, Tis were merged and referred to as “Ta/Tis”. T1, T1a, T1b, T1 NOS were merged and collectively referred to as “T1”. T2, T2a, T2b, T2 NOS were merged and collectively referred to as “T2”. T3, T3a, T3b, T3c, T3 NOS were merged and collectively referred to as “T3”. T4, T4a, T4b, T4 NOS were merged and collectively referred to as “T4”. The surgery codes 10 (local tumor destruction), 20 (local tumor excision), and 30 (partial cystectomy) were merged and collectively referred to as “local procedure/ partial cystectomy”. Surgical codes 50 (simple/total/complete cystectomy), 60 (complete cystectomy with reconstruction), and 70 (pelvic exenteration) were combined, and collectively referred to as “complete cystectomy”. Surgical codes 80 (cystectomy, NOS) and 90 (surgery, NOS) were combined and collectively referred to as “surgery, NOS”. Detailed SEER database surgery codes are available at (https://seer.cancer.gov/manuals/2018/appendixc.html). Cases diagnosed at autopsy or that could have 0 days of follow-up were excluded all analyses except for incidence and mortality calculations.

### 2.3. Statistical Analysis

All statistical analysis was carried out using the IBM SPSS Statistics software package (version 25, International Business Machines Corporation, Armonk, NY, USA). The significance of incidence and mortality trends were calculated using linear regression analysis. Differences in demographic and clinical characteristics between CCA and non-CCA were determined using the Pearson’s chi-square test. Median survival times were determined using the Kaplan–Meier method, and the significance was determined using the log-rank test. Multivariable analyses of overall survival were conducted using the Cox proportional hazards ratios (HR) model. Corresponding HR and 95% confidence intervals (CI) were estimated from the model. Two-tailed *p*-values < 0.05 were considered statistically significant. 

## 3. Results

### 3.1. Incidence and Mortality of CCA 

To assess recent trends in the incidence and mortality of CCA, we queried all cases of CCA from 2004 to 2015 in the SEER Program database. Over this period, the age-adjusted the incidence of CCA was 0.087 individuals per 1,000,000 (Supplementary Table S1). Our analysis suggested a downward trend in incidence over this period—a shift from 0.062 per 1,000,000 in 2004 to 0.057 per 1,000,000 individuals in 2015 with an annual decrease rate of 0.003. However, this trend was non-significant (*p* = 0.178, Supplementary Figure S1A). We further assessed incidence separated by gender (Supplementary Table S1). The incidence of CCA among female and males were similar, with a slight female predominance—0.091 and 0.084 per 10,000,000 for females and males respectively from 2004 to 2015 (Supplementary Table S1). 

The mortality rate from 2004–2015 was 0.064 individuals per 1,000,000 with an increasing trend of 0.002 per year. This trend was also non-significant (*p* = 0.477, Supplementary Figure S1B, Supplementary Table S1). When separated by gender, male with CCA had higher mortality rate of 0.074 compared to 0.058 in females per 1,000,000 individuals (Table S1). 

### 3.2. Demographics and Clinical Characteristics

To compare demographical and clinical characteristics of CCA to non-CCA cancers of the urinary bladder, we utilized cases of malignant carcinomas of the urinary bladder from 2004, when AJCC 6th staging information became available, to 2015, the most recent data available at time of analysis. We obtained 205,197 cases of malignant urinary bladder carcinoma. Of these, 91 cases (0.04%) were identified as CCA. The median follow-up time was 19 months with 45 deaths in these CCA patients. Amongst 205,106 cases of non-CCA patients, the median follow-up time was 23 months, with 68,951 recorded deaths. The median age at diagnosis of CCA was 70 years old and median age at diagnosis was 72 years old in non-CCA patients.

The demographical and clinical characteristics of the patient population are summarized in Table 1. Our results showed that CCA patients were more likely to be younger age (<60 years of age; *p* = 0.005), female (*p* < 0.001) and black (*p* = 0.001) than non-CCA patients. The larger proportion of female patients is consistent with our incidence analysis. CCA patients also had higher grade (*p* < 0.001), higher AJCC 6th staging (*p* < 0.001) including TNM staging (*p* values for T, N, M stage were *p* < 0.001, *p* < 0.001 and *p* < 0.001, respectively). The primary site of tumor location was significantly different between CCA and non-CCA patients (*p* < 0.001); CCA patients were more likely to have tumors in the trigone of bladder, bladder neck and urachus, whereas non-CCA tumors appeared mostly in the lateral wall of bladder. As expected with more advanced tumor staging, CCA patients showed higher likelihood of brain (*p* < 0.001) and liver (*p* = 0.028) metastasis. However, very few cases with metastasis were available; only a single case was available for brain metastasis and two cases for liver metastasis. Furthermore, our data showed that non-CCA patients were more likely to receive fewer radical treatments such as local procedure or partial cystectomy, while more CCA patients received complete cystectomies (*p* < 0.001). The majority of non-CCA patients did not receive radiation, while a greater number of CCA patients received beam radiation (*p* < 0.001). 

### 3.3. Survival

**Figure 1:**
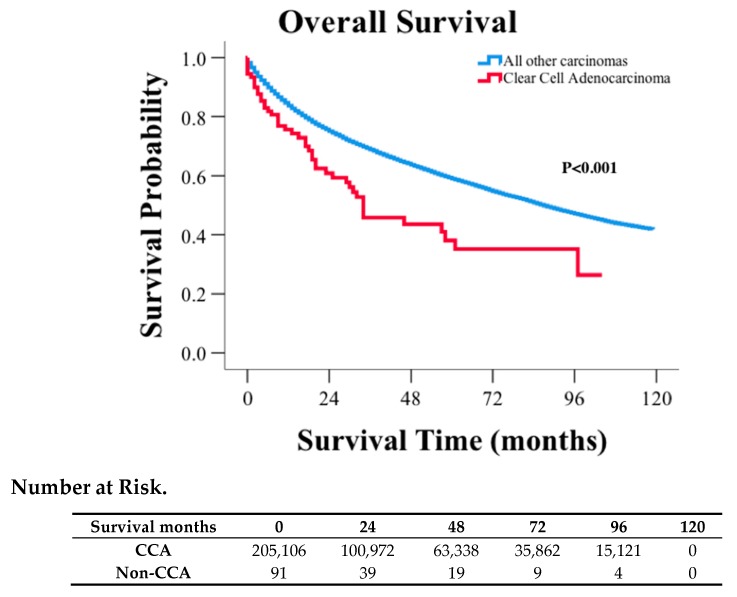
Kaplan–Meier curve and risk table of clear cell adenocarcinoma in comparison to other carcinomas of the urinary bladder.

The median survival for CCA patients was 34 months with 5- and 10-year survival rates of 41%, 30%, respectively. The median survival for non-CCA patients was 87 months, with corresponding 5- and 10-year survival rates of 61% and 44%, respectively (Figure 1, *p* < 0.001). Using multivariable analysis accounting for age, sex, race, AJCC 6th stage, tumor grade, surgery, and radiation treatment, survival for CCA patients was no longer significantly poorer than non-CCA patients (HR: 0.93; 95% CI: 0.69–1.255; *p* = 0.636, Supplementary Table S2 left half). However, when staging was removed from same multivariable analysis, CCA survival remained significantly shorter than non-CCA patients (HR: 1.435, 95% CI: 1.064–1.936, *p* = 0.018, Supplementary Table S2 right half). Therefore, the histological subtype CCA is not an independent prognostic factor for survival, but instead, it is the more advanced staging in CCA patients accounts for the survival difference between CCA and non-CCA patients.

To further confirm our finding that the worse prognosis is attributable for the higher staging, we stratified our CCA cases according to AJCC 6th staging and compared survival in patients with non-muscle invasive (AJCC 6th stage 0 and I), muscle-invasive (AJCC 6th stage II and III) and metastatic (AJCC 6th stage IV) pathology. As suspected, when stratified by non-muscle invasive, muscle-invasive, and metastatic cases, the survival durations were no longer significantly different between CCA and non-CCA cases (Table 2, *p* = 0.654, *p* = 0.653, *p* = 0.091 respectively). 

**Figure 2:**
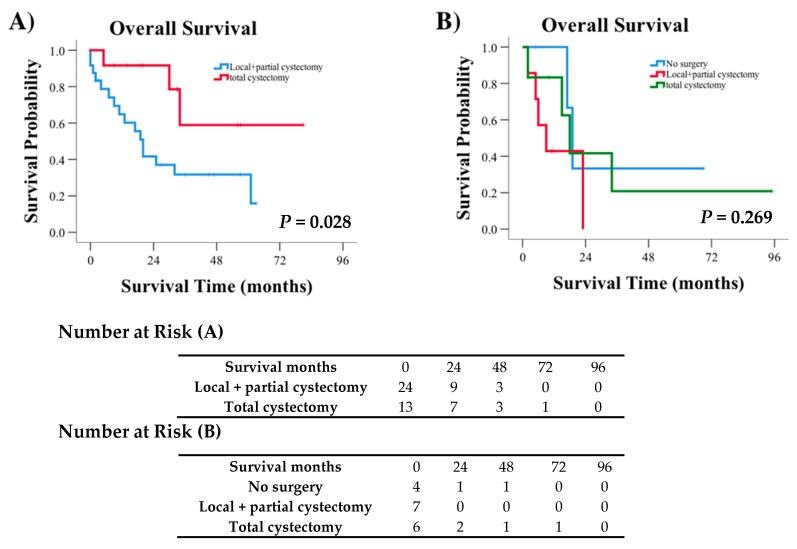
Kaplan–Meier curves and risk tables demonstrating survival for (**A**) muscle invasive cases of CCA defined by AJCC 6th stage II and III and (**B**) metastatic CCA cases defined by AJCC 6th stage IV.

Moreover, when surgical procedure was assessed in each subgroup of patients stratified by staging, a significant difference in the survival of muscle-invasive CCA patients was observed. Patients receiving total cystectomy showed significantly greater survival probability than those receiving local procedures or partial cystectomy (*p* = 0.028, Figure 2A). However, for metastatic cases, no survival difference was observed based on surgical treatment received (*p* = 0.269, Figure 2B). Survival comparisons for non-muscle invasive cases were unable to be conducted due to the large number of censored events, i.e., patients that did not die during the follow-up period. 

## 4. Discussion

Using the SEER program database, we documented incidence, mortality, demographics, and survival on a rare subtype of urinary bladder cancer. We identified that CCAs were more commonly associated with younger age, higher grade, female gender, black ethnicity, and have a higher risk of brain and liver metastasis. Although it was not present in any of the cases reported in the SEER program database, bone metastasis in CCAs has been reported in several previously published case reports [28,29]. The most common location of CCA identified from our study was from trigone and bladder neck. This finding is consistent with previous reviews that also documented these as common tumor locations [21,28]. More importantly, our study showed a poorer prognosis of CCAs compared to all other carcinomas of the urinary bladder attributable to the higher tumor staging of the CCA cases. The poorer prognosis was irrespective of age, sex, race, grade, surgery and radiation treatment. In muscle invasive cases of CCA, type of surgical treatment was a significant factor in determining survival—There was improved survival when treated with complete cystectomies, which is consistent with standard of care for carcinomas of the urinary bladder [30]. 

The capability for glycogen to enhance tumor survival in adverse conditions may result in a faster invasion of CCA, hence, higher staging at diagnosis. Glycogen stores provide an excess glucose supply that can be utilized in the hypoxic conditions of tumor microenvironment [7]. The glycogen breakdown also generates nucleotides critical for cell proliferation such as NAPDH, an essential reducing agent, through the pentose phosphate pathway [7]. Furthermore, the glycogen shunt has been proposed to sustain the Warburg effect, a phenomenon that causes cells to use glucose in glycolysis instead of oxidative phosphorylation even in presence of plentiful oxygen in cells [31]. During periods of decreased glucose availability, the glycogen shunt sustains the production of glycolytic intermediates and ATP through the Warburg effect, hence maintaining tumor growth in nutrient deprived conditions [5]. 

Recently, the glycogen debranching enzyme amylo-α-1, 6-glucosidase, 4-α-glucanotransferase (AGL) was shown to have tumor suppressor functions in a model of urothelial bladder cancer [32]. Loss of AGL increased tumor growth in vitro and in xenografted tumors accompanied by an increase in abnormal glycogen structures (limit dextrin) and decrease in normal glycogen. The study also showed an increase in aerobic glycolysis and increased lactate, consistent with a shift towards the Warburg effect. Similar to our results, patients with reduced AGL expression was also associated with a decrease in overall survival, but was no longer predictive of survival when examined in a multivariate model that included age, sex, stage, and grade [32]. The similarities of our findings in CCA suggest that the manipulation of glycogen accumulations in urothelial bladder tumors may induce characteristics that mimic CCA.

While most urinary bladder cancers are male predominant [33], it was an interesting finding that CCA seemed to have a female predominance. The higher proportion of female patients supports a possible mullerian origin of CCA which has been previously proposed due to its association with endometriosis and histological resemblance to clear cell cancers of female genital tract [19,34]. Moreover, it is well known that females with urinary bladder cancers are generally diagnosed with more advanced disease and have poorer prognosis than males [33,35]. However, our findings suggested that it was CCA males instead who had higher mortality than females. Collectively, the gender disparity between CCA and other urinary bladder cancers suggest that CCA is an entity with differing characteristics to other urinary bladder cancers. More mechanistic and clinical studies are needed to improve our understanding of how gender and its associated factors relate to CCA pathology and prognosis. 

At this time, no tailored therapy exists for CCA. Patients typically undergo some form of surgical resection such as transurethral resection, total cystectomy, partial cystectomy or radical surgery accompanied by chemotherapy and/or radiation [30]. Our study suggests that those with muscle invasive disease had survival benefit from total cystectomy rather than partial cystectomy, although prospective studies are needed to confirm these findings. Further understanding of cancer glycogen metabolism may help us with new avenues of tailored disease treatment. No information with regards to chemotherapy treatment was included in this manuscript due to a lack of reliable data in the SEER program database at this time. 

## 5. Conclusions

As the first large-scale study to date, we assessed the incidence, mortality, demographical/clinical characteristics, and survival of CCA, a rare, glycogen-rich variant of urinary bladder cancer. We found a poorer prognosis of CCAs compared to all other carcinomas of the urinary bladder that was attributable to the higher staging of these tumors. However, the limitations of the study include the retrospective study design, small number of cases of interest (i.e., CCA) in comparison to control cases (i.e., non-CCA), and reliability of the SEER program database. Additional prospective clinical studies are needed to confirm these findings. Mechanistic studies that assess signaling pathways linking glycogen and rate of tumor growth would be beneficial for improving the understanding of the link between glycogen and poorer patient prognosis, and help to identify novel, targeted therapies for these glycogen-rich cancers. 

## Figures and Tables

**Table 1:** Demographical and clinical characteristics comparing clear cell adenocarcinoma to other carcinomas of the urinary bladder.

		Clear Cell Adenocarcinoma (*N* = 91)		Non-Clear Cell Adenocarcinoma (*N* = 205,106)		
		Count	%	Count	%	*p*-Value
Age	0–60	27	29.7	37,649	18.4	0.005
	61+	64	70.3	167,457	81.6	
Sex	Female	54	59.3	49,241	24.0	<0.001
	Male	37	40.7	155,865	76.0	
Race	White	77	84.6	182,492	89.0	0.001
	Black	13	14.3	11,519	5.6	
	Other	1	1.1	8588	4.2	
	Unknown	0	0.0	2507	1.2	
Tumor primary site	Trigone of bladder	9	9.9	12,765	6.2	<0.001
	Dome of bladder	4	4.4	7213	3.5	
	Lateral wall of bladder	6	6.6	41,041	20.0	
	Anterior wall of bladder	5	5.5	4334	2.1	
	Posterior wall of bladder	7	7.7	18,819	9.2	
	Bladder neck	10	11.0	6354	3.1	
	Ureteric orifice	4	4.4	7820	3.8	
	Urachus	1	1.1	310	0.2	
	Overlapping lesion of bladder	13	14.3	21,112	10.3	
	Bladder, NOS	32	35.2	85,338	41.6	
Grade	Grade I	0	0.0	23,684	11.5	<0.001
	Grade II	5	5.5	48,123	23.5	
	Grade III	22	24.2	35,849	17.5	
	Grade IV	25	27.5	59,477	29.0	
	Unknown	39	42.9	37,973	18.5	
AJCC 6th stage	Stage 0	2	2.2	105,545	51.5	<0.001
	Stage 1	22	24.2	46,332	22.6	
	Stage 2	28	30.8	23,463	11.4	
	Stage 3	9	9.9	8157	4.0	
	Stage 4	17	18.7	14,012	6.8	
	Unknown	13	14.3	7597	3.7	
T stage	Tis/Ta	2	2.2	105,545	51.5	<0.001
	T0	0	0.0	91	0.0	
	T1	26	28.6	49,221	24.0	
	T2	35	38.5	28,776	14.0	
	T3	7	7.7	8046	3.9	
	T4	11	12.1	7713	3.8	
	Unknown	10	11.0	5714	2.8	
N stage	N0	70	76.9	189,973	92.6	<0.001
	N1	2	2.2	3994	1.9	
	N2	6	6.6	3806	1.9	
	N3	1	1.1	166	0.1	
	Unknown	12	13.2	7167	3.5	
M stage	M0	75	82.4	193,071	94.1	<0.001
	M1	11	12.1	7565	3.7	
	Unknown	5	5.5	4470	2.2	
Bone metastasis ^a^	No	42	97.7	102,083	97.0	0.697
	Yes	0	0.0	1432	1.4	
	Unknown	1	2.3	1698	1.6	
Brain metastasis ^a^	No	41	95.3	103,393	98.3	<0.001
	Yes	1	2.3	122	0.1	
	Unknown	1	2.3	1698	1.6	
Liver metastasis^a^	No	40	93.0	102,600	97.5	0.028
	Yes	2	4.7	926	0.9	
	Unknown	1	2.3	1687	1.6	
Lung metastasis ^a^	No	41	95.3	102,153	97.1	0.771
	Yes	1	2.3	1327	1.3	
	Unknown	1	2.3	1733	1.6	
Type of surgical procedure	No surgery	8	8.8	15,265	7.4	<0.001
	Local procedure/partial cystectomy	60	65.9	170,325	83.0	
	Complete cystectomy	22	24.2	18,327	8.9	
	Surgery NOS	0	0.0	504	0.2	
	Unknown if surgery performed	1	1.1	685	0.3	
Type of radiation ^b^	None	67	85.9	160,440	94.6	<0.001
	Beam radiation	9	11.5	7485	4.4	
	Other radiation	1	1.3	219	0.1	
	Unknown if radiation received	1	1.3	1389	0.8	

Bolded are statistically significant *p*-values when comparing between clear cell adenocarcinoma to other carcinomas of the urinary bladder. NA—not applicable. ^a^ Variable only available for cases diagnosed after 2010. ^b^ Variable only available for cases diagnosed before 2013.

**Table 2:** Survival comparison between clear cell adenocarcinoma and other urinary bladder cancers stratified by stage.

		Median Survival	95% Confidence Interval		
			Lower Bound	Upper Bound	*p*-Value
Non-muscle invasive (Stage 0–1)	Non-CCA	119			0.654
	CCA				
Muscle invasive (Stage 2–3)	Non-CCA	25	24.263	25.737	0.653
	CCA	32	16.308	47.692	
Metastatic (Stage 4)	Non-CCA	9	8.736	9.264	0.091
	CCA	18	13.315	22.685	

## Supplementary Materials

The following are available online at https://www.mdpi.com/2077-0383/9/1/138/s1, Table S1: Incidence and mortality of clear cell carcinoma of the urinary bladder from 2004–2015 per million population, Table S2: Multivariable analysis of survival for all urinary bladder patients, Figure S1: (**A**) Incidence of clear cell adenocarcinoma per million individuals in the US population. (**B**) Mortality of clear cell adenocarcinoma per million individuals in the US population.
