# Supplementary material for: Clear Cell Adenocarcinoma of the Urinary Bladder Is a Glycogen-Rich Tumor with Poorer Prognosis
Source: J Clin Med. 2020 Jan 3;9(1):138. doi: 10.3390/jcm9010138 (PMC7019566; doi:10.3390/jcm9010138)
Supplement: Supplementary file 1 [file jcm-09-00138-s001.pdf]

Journal of
Clinical Medicine

MDPI

Table S1. Incidence and mortality of clear cell carcinoma of the urinary bladder from 2004–2015 per million population.

	Overall (95% Confidence Interval)	Female (95% Confidence Interval)	Male (95% Confidence Interval)
Incidence	0.087 (0.069–0.107)	0.091 (0.068–0.119)	0.084 (0.059–0.116)
Mortality	0.064 (0.049–0.081)	0.058 (0.040–0.082)	0.074 (0.050–0.106)

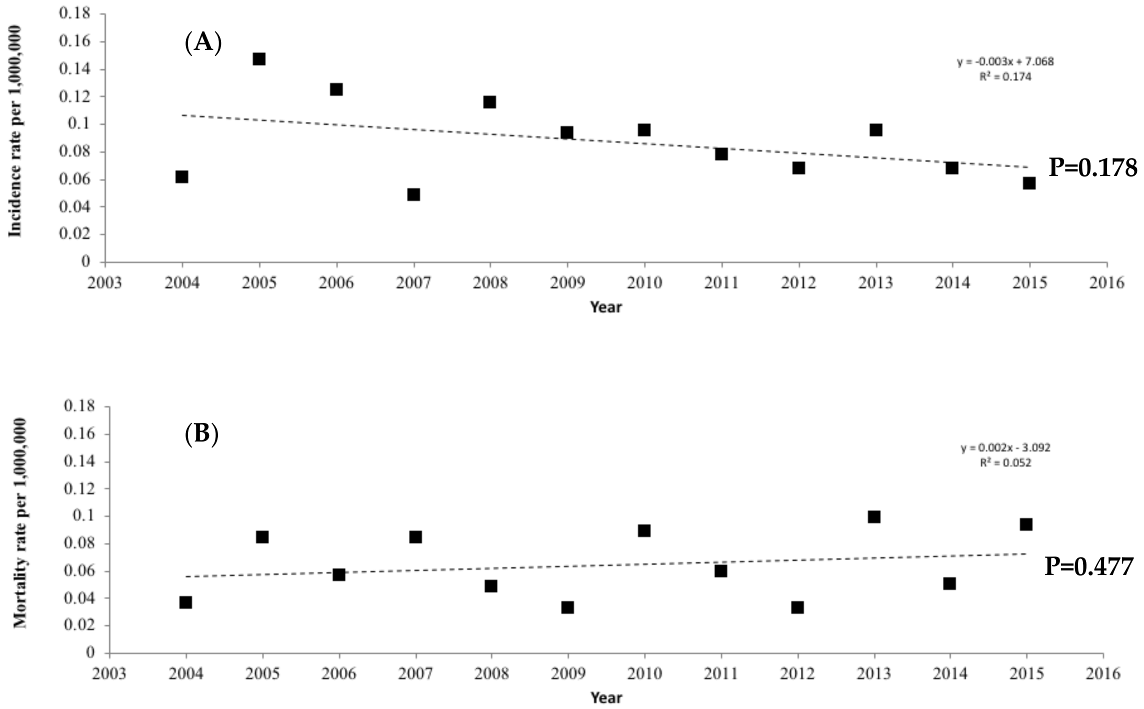

Figure S1. (**A**) Incidence of clear cell adenocarcinoma per million individuals in the US population. (**B**) Mortality of clear cell adenocarcinoma per million individuals in the US population.

Table S2. Multivariable analysis of survival for all urinary bladder patients.

		With Staging				Without Staging			
	**Reference**	***p*-Value**	**HR**	**95% CI**		***p*-Value**	**HR**	**95% CI**	
				**Lower**	**Upper**			**Lower**	**Upper**
Clear cell adenocarcinoma	All other carcinomas	0.636	0.930	0.690	1.255	0.018	1.435	1.064	1.936
Age		<0.001	1.056	1.055	1.057	<0.001	1.055	1.054	1.056
Sex	Female	0.009	1.025	1.006	1.043	0.011	0.977	0.959	0.995
Race	White	<0.001				<0.001			
Black		<0.001	1.306	1.265	1.347	<0.001	1.434	1.390	1.480
Other		<0.001	0.837	0.803	0.873	<0.001	0.854	0.819	0.891
Unknown		<0.001	0.357	0.310	0.412	<0.001	0.314	0.272	0.362
Grade	Grade I	<0.001				<0.001			
Grade II		0.002	1.054	1.02	1.089	<0.001	1.126	1.09	1.164
Grade III		<0.001	1.342	1.298	1.388	<0.001	2.158	2.09	2.228
Grade IV		<0.001	1.274	1.232	1.317	<0.001	2.105	2.04	2.172
Unknown		<0.001	1.195	1.154	1.237	<0.001	1.436	1.387	1.486
AJCC6th stage	Stage 0	<0.001				-			
Stage 1		<0.001	1.495	1.461	1.53		-	-	-
Stage 2		<0.001	3.507	3.412	3.605		-	-	-
Stage 3		<0.001	4.904	4.721	5.095		-	-	-
Stage 4		<0.001	11.626	11.276	11.987		-	-	-
Unknown		<0.001	2.676	2.578	2.777		-	-	-
Surgery	None	<0.001				<0.001			
Local + partial cystectomy		<0.001	0.711	0.691	0.732	<0.001	0.533	0.519	0.547
Total cystectomy		<0.001	0.407	0.392	0.423	<0.001	0.841	0.812	0.871
Surgery NOS		<0.001	0.641	0.558	0.737	<0.001	0.777	0.677	0.893
Unknown		<0.001	0.634	0.543	0.74	<0.001	0.532	0.456	0.621
Radiation	None	<0.001				<0.001			
Beam radiation		0.883	0.998	0.968	1.029	<0.001	2.182	2.12	2.246
Other radiation		0.595	0.958	0.818	1.122	<0.001	1.864	1.592	2.182
Unknown		<0.001	1.287	1.184	1.399	<0.001	1.595	1.467	1.733

Not applicable.
